# Spatio-Temporal Patterns of Pancreatic Cancer Cells Expressing CD44 Isoforms on Supported Membranes Displaying Hyaluronic Acid Oligomers Arrays

**DOI:** 10.1371/journal.pone.0042991

**Published:** 2012-08-14

**Authors:** Thomas Kaindl, Harden Rieger, Lisa-Mareike Kaschel, Ulrike Engel, Anja Schmaus, Jonathan Sleeman, Motomu Tanaka

**Affiliations:** 1 Physical Chemistry of Biosystems, Institute of Physical Chemistry, University of Heidelberg, Heidelberg, Germany; 2 Institute of Toxicity and Genetics, Karlsruhe Institute of Technology, Karlsruhe, Germany; 3 Medical Faculty Mannheim of the University of Heidelberg, Centre for Biomedicine and Medical Technology Mannheim (CBTM), Mannheim, Germany; 4 Nikon Imaging Center, University of Heidelberg, BIOQUANT, Heidelberg, Germany; Cornell University, United States of America

## Abstract

In this paper, we designed a quantitative model of biological membranes by the deposition of planar lipid membranes on solid substrates (called supported membranes), and immobilized biotinylated oligomers of hyaluronic acid (oligo-HA, 6–8 disaccharide units in length) to the membrane surface via neutravidin cross-linkers. By controlling the self-assembly of biotinylated lipid anchors, the mean distance between the oligo-HA molecules on the membrane could be controlled to nm accuracy. The adhesion and motility of pancreatic adenocarcinoma cells expressing different splice variants of the HA-binding cell surface receptor CD44 on these surfaces were investigated quantitatively. The combination of label-free, time-lapse imaging of living cells and statistical analysis suggests that the static morphology (global shape and cytoskeleton remodeling) of cells, their stochastic morphological dynamics, and the probability of directed motion reflect the metastatic behaviour of the cancer cells.

## Introduction

In recent years, CD44 has emerged as an important mediator of cell-matrix interactions that integrates the signaling activity of growth factor receptors through its co-receptor function [1]. Expression of the CD44 gene gives rise to a family of transmembrane glycoproteins whose molecular weight is extremely heterogeneous (MW 80–200 kDa) due to variable N- and O-linked glycosylation and alternative splicing [2], [3], [4]. In particular, the insertion of up to 10 variant exons during alternative splicing of the CD44 transcript introduces extensive variability into the extracellular membrane proximal region of the protein [5], [6], [7]. These variant exon-containing isoforms are termed CD44v, in contrast to CD44s that does not contain these variant exons.

The interaction of CD44 with the extracellular matrix glycosaminoglycan hyaluronan (HA) is the most intensively studied interaction of the CD44 protein [8]. This interaction is regulated at a number of levels, including glycosylation [4] and the clustering of CD44 that is promoted by the inclusion of variant exon-encoded sequences [9]. HA is synthesized as a high molecular weight polymer comprised of alternating subunits of N-acetylglucosamine and glucuronic acid [10]. During tumor growth and inflammation, degradation of HA can be enhanced, resulting in the accumulation of small HA oligosaccharides that exert biological activities not exhibited by high molecular weight HA [11]. Two HA binding motifs in the extracellular portion of the CD44 protein mediate its interaction with HA [12]. CD44 binds to the minimum of a HA hexasaccharide [13], and signaling via CD44 can be regulated by the size of HA [1].

HA and CD44 have both been implicated in the regulation of tumor growth and metastasis [4], [14], [15]. Accumulation of HA is associated with poor patient prognosis and has been suggested to increase tumor proliferation, invasion and angiogenesis amongst others [8]. Additionally, expression of different isoforms of CD44 has been related to poor prognosis in a number of different tumor types [14], and studies in animal models have provided evidence for a functional role of CD44 isoforms in metastasis [16]. Importantly, the interaction between CD44 and HA has been associated with tumor growth and metastasis [17], [18]. However, contradictory data also exist. In some contexts accumulation of HA decreases tumorigenicity [19], [20], [21], [22], while the expression of hyaluronidases, enzymes that degrade HA, can correlate with tumor progression [23]. Similarly expression of some isoforms of CD44 in particular types of cancer can correlate with good prognosis [15], and suppress metastasis in animal models [24]. Together these observations suggest that a better understanding of how CD44 interacts with HA is required to explain the relevance of these complex interactions to tumor progression and metastasis, which in turn will identify new routes for therapeutic intervention.

The rat pancreatic carcinoma model BSp73 [25] provides a useful model for analyzing both the metastasis-promoting functions of CD44 as well as the interaction between CD44 and HA. The BSp73AS cell line (called 1AS in the following text) is weakly metastatic, expresses CD44s but only very low endogenous levels of CD44 variants, and binds poorly to immobilized HA [26]. Transfection of these cells with CD44v4-v7, a splice variant found in highly metastatic cells, produced the cell line ASpSV14 which is highly metastatic in rat models [16]. Expression of the CD44v4-v7 protein also promotes the binding of ASpSV14 cells to HA through regulated clustering of the CD44v4-v7 protein [9]. A R44L point mutation in the N-terminal HA binding motif of the CD44v4-v7 protein renders the protein unable to bind to HA, whereas a K162A, R166A double point mutation in the other HA binding motif of CD44v4-v7 results in a reduced HA binding capacity compared to the wild-type CD44v4-v7 protein [26]. Accordingly, 1AS cells ectopically expressing the R44L CD44v4-v7 protein (AS-R44 cells) do not bind HA, while 1AS cells ectopically expressing the K162A, R166A CD44v4-v7 protein (AS-K162R166 cells) show reduced binding to HA compared to ASpSV14 cells [26].

Using these four cell lines, we designed experiments to examine the interaction of CD44 with precisely spatially ordered HA of defined length (6–8 disaccharide units). Specifically, the adhesion and motility of rat pancreatic cancer cells expressing different CD44 isoforms were studied on defined lateral densities of HA. Instead of non-specific physisorption or covalent grafting of oligo-HA molecules on plastic substrates, we anchored biotinylated oligo-HA molecules via neutravidin cross-linkers to the surface of planar lipid membranes (so-called “supported membranes” [27], [28]) that incorporated biotinylated lipid anchors. This allowed the non-specific adhesion of cells by means of long-range Van der Waals attraction to be minimized, and the average distance between anchored HA molecules to be precisely controlled within nm accuracy [29]. By employing a combination of time-lapse imaging of the cells with label-free micro-interferometry (RICM) [30], [31] and statistical analysis [32], we could quantitatively evaluate the strength of cell adhesion, stochastic dynamics of cell morphology, and persistence and speed of cell motility.

## Materials and Methods

### Materials

1,2-Dioleoyl-sn-Glycero-3-phosphocholine (DOPC) and 1,2-dioleoyl-sn-glycero-phospho-ethanolamine-3-N-(cap biotinyl) (biotin-DOPE) were purchased from Avanti Polar Lipids (Alabaster, AL, USA), and deglycosylated neutravidin from Invitrogen (Karlsruhe, Germany). The far-red fluorescent DNA dye DRAQ5 from biostatus limited (Leicestershire, UK) was used to stain the cell nuclei, and Alexa Fluor 488 phalloidin (Invitrogen, Karlsruhe, Germany) was used for the visualization of the actin cytoskeleton. Adhesion assays were performed in bottomless plastic fluidic channels (µ-slide I) from ibidi (Munich, Germany) sealed with microscopic grade 25×75 mm^2^ glass slides from Menzel (Braunschweig, Germany). All the other chemicals of p.A. quality were purchased from Sigma-Aldrich (Neu-Ulm, Germany), if not otherwise specified. Throughout this study, de-ionized ultrapure water (Genpure, TKA Niederelbern, Germany) was used for preparation of all buffer solutions.

**Figure 1:**
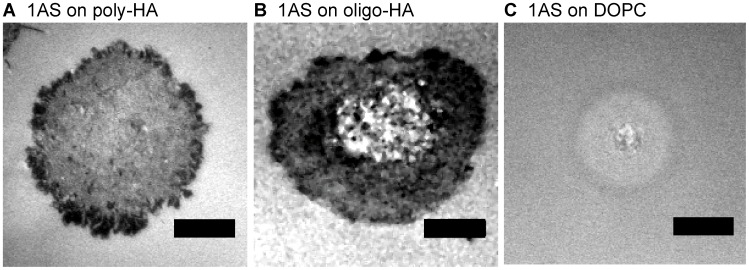
Cell adhesion on poly-HA and oligo-HA substrates. Micro-interferometry images of 1AS cells on supported membranes displaying (A) poly-HA and (B) oligo-HA at an average distance of <*d*> ∼ 5.5 nm at *t*  =  2 h. Scale bar: 8 µm.

**Figure 2:**
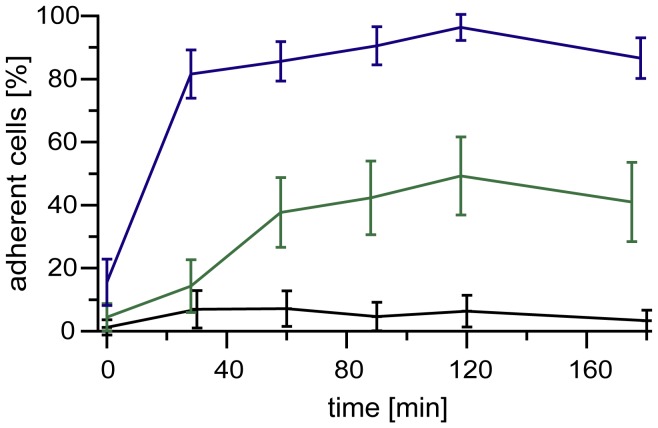
The impact of the average distance between oligo-HA molecules on adhesion of 1AS cells. The fraction of 1AS cells adhered to the indicated supported membranes plotted as function of time. The three membranes studied were pure DOPC membranes (black line), and membranes displaying oligo-HA at <*d*> ∼ 11 nm (green line) and <*d*> ∼ 5.5 nm (blue line).

### HA Sample Preparation

Hyaluronic acid polymer (poly-HA) was purchased from Sigma-Aldrich (Neu-Ulm, Germany). Hyaluronic acid oligomer (oligo-HA, GE Healthcare, Heidelberg, Germany) was prepared by enzymatic degradation with bovine testis hyaluronidase [33] and fractionation with an ÄKTA purifier (GE Healthcare, Heidelberg, Germany) to obtain oligomers of 6–8 disaccharide units in length. For the immobilization to the membrane surface, both poly- and oligo-HAs were modified by coupling with (+)-biotin hydrazide (Sigma-Aldrich, Neu-Ulm, Germany) [34], [35].

### HA Surface Preparation

Before bonding, glass substrates were cleaned by using a modified RCA protocol [36]. Specifically, the substrates were sonicated for 5 min in acetone, ethanol, methanol, and water, then immersed in a solution of H_2_O_2_ (30%) / NH_4_OH (30%) / H_2_O (1∶1:5 by volume) and sonicated for 5 min at room temperature before soaking them for another 30 min at 60°C. Afterwards, they were intensively rinsed with water, dried at 70°C, and stored in a vacuum chamber. Lipid mixtures with different molar ratios of biotin-DOPE were first exposed to a nitrogen stream, and then kept in a vacuum chamber at 25°C for 12 h. After suspending the dry lipid films in de-ionized water, small unilamellar vesicles (SUVs) were prepared by sonication with a tip sonicator (Misonix, New York, USA) for 30 min at 1.0 W. Supported membranes were prepared by deposition of SUV suspensions on cleaned substrates. After 20 min of incubation, the chambers were intensively rinsed with de-ionized water to remove the remaining vesicles. The average distance between lipid anchors (biotin-DOPE) and hence the distance between HA molecules can be estimated from the molar fraction of biotin-DOPE χ_b-DOPE_ and the area per lipids (*A*_lipid_ ∼ 60 Å^2^ [37]), as 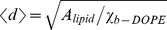. In the next step, the membrane was incubated in neutravidin solution (1 µg/ml, in de-ionized water) for 20 min. After intensive rinsing, an aqueous solution of biotinylated HA (0.4 mg/ml) was injected into each chamber of the glass slides and incubated for 20 min. Finally, the samples were washed with pre-warmed cell culture medium and kept at 37°C prior to the adhesion experiments.

**Table 1:** Shape descriptors of adhesion area.

Sample	Projected area [µm^2^]	Elongation	Circularity
1AS on poly-HA	330±190	0.84±0.10	0.59±0.17
1AS on oligo-HA	420±90	0.75±0.13	0.58±0.06

Comparison of projected area per adhered cell, elongation, and circularity of 1AS cells on supported membranes functionalized with poly-HA and oligo-HA at <*d*> ∼ 5.5 nm and *t*  =  2 h. Standard deviation is given for mean values of nine cells for each condition.

**Figure 3:**
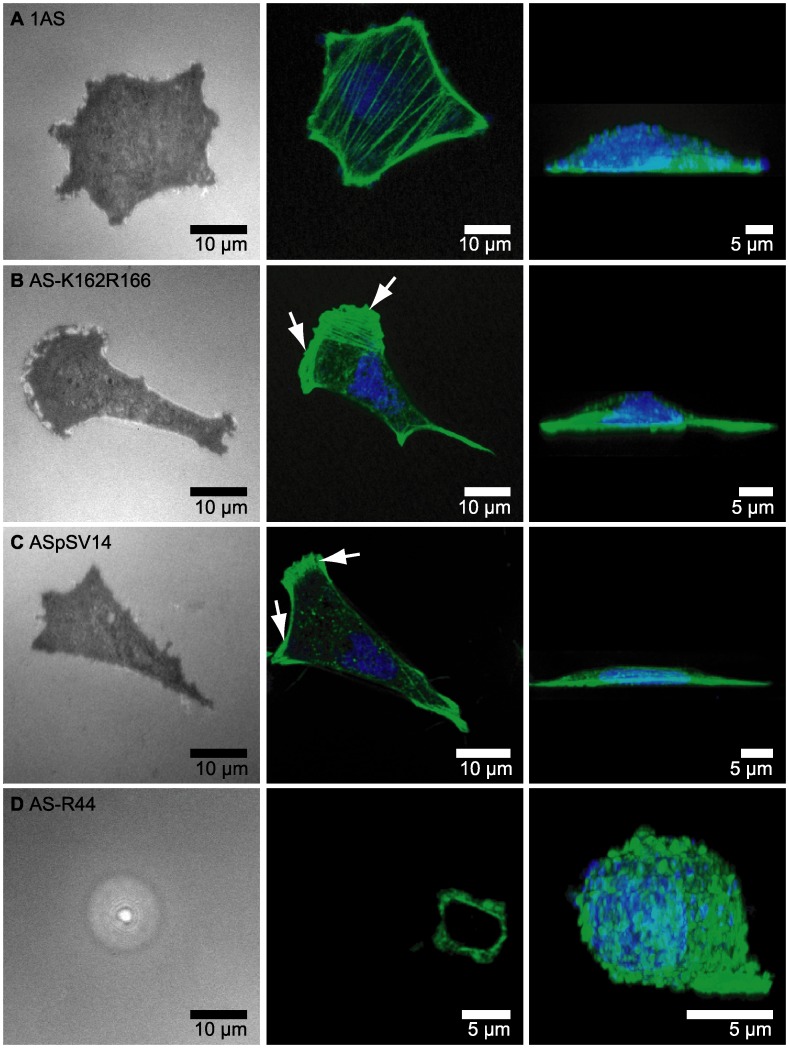
Binding of cancer cells on oligo-HA tethered supported membranes. RICM images (left) and confocal fluorescence images (middle and right) of (A) 1AS cells (B) AS-K162R166 cells (C) ASpSV14 cells, and (D) AS-R44 cells incubated on supported membranes displaying oligo-HA at <*d*> ∼ 5.5 nm for 4 h. After fixation, DNA and actin were stained with DAPI and Alexa 488 phalloidin.

### Cell Adhesion Experiments

1AS, ASpSV14, AS-R44 and AS-K162R166 cells were cultivated as previously described [26], [38]. Cells were harvested by trypsination and subsequently re-suspended in pre-warmed RPMI 1640 at a density of 1.75×10^7^ cells/ml. A 0.2 ml portion of cell suspension was injected into each chamber of the glass slides, and maintained at 37°C and 5 % CO_2_. Non-invasive live cell imaging was performed using reflection interference contrast microscopy (RICM) on an Axiovert 200 inverted microscope (Carl Zeiss, Göttingen, Germany) equipped with a PlanNeofluar 63×/1.25 Antiflex oil-immersion objective. To calculate the area of cell-membrane contact, images were recorded with an Orca ER CCD camera (Hamamatsu Photonics, Herrsching, Germany). After the subtraction of the background, the images were binarized to determine the edge of the adhesion zone. The fraction of adhered cells was determined by normalizing the cells observed by RICM (adhered cells) with total cells in the corresponding phase contrast images. Cells for time-dependent data acquisition were also incubated in RPMI 1640 cell medium at 37°C and 5 % CO_2_. Images were taken at a fixed position with time intervals of 10 min and 15 min for 20 h. After extraction of the cell contour line from each binary picture, the center of mass was extracted and used for the documentation of cell displacement and contour line analysis. Both values, adhesion area *A* and cell contour line length *L*, were used for the calculation of the dimensionless circularity 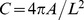. The elongation factor was further deduced by the ratio of perpendicular minor to major axes, and thus also results in a dimensionless range between 1 and 0.

**Figure 4:**
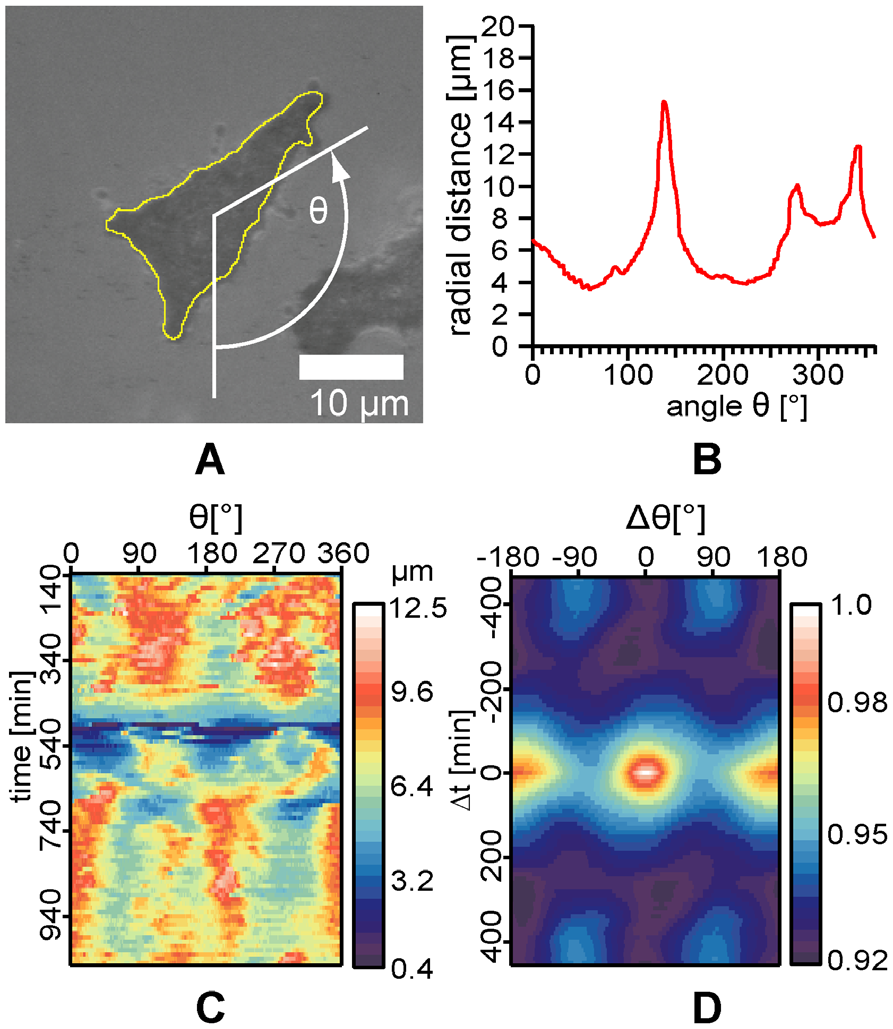
Time evolution of cell morphology. (A) A snap shot of an ASpSV14 cell on an oligo-HA-functionalized membrane (<*d*> ∼ 5.5 nm, *t*  =  9 h) captured by RICM. The peripheral edge of the cell was determined by the contrast in pixel intensity. (B) The amplitude of the fluctuation amplitude 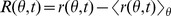 plotted as a function of *θ*. 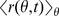 is the mean radial distance over *θ*  =  0–360°. (C) The amplitude map as a function of angle *θ* over time (*t*  =  140–1000 min). (D) The autocorrelation corresponding to the amplitude map in panel (C).

## Results

### Adhesion of 1AS to Poly- and Oligo-HA Tethered to Supported Membranes

To compare the HA binding characteristics of 1AS cells and their derivatives, we first examined the ability of 1AS to adhere to HA tethered to supported membranes. Figure 1 shows representative RICM images of 1AS cells binding for 2 h to poly-HA and oligo-HA tethered at an average intermolecular distance of <*d*> ∼ 5.5 nm. In this study, the average intermolecular distance <*d*> can be controlled by the molar concentration of freely diffusive anchoring molecules in the supported membrane. As presented in the Figure 1, 1AS cells showed a pronounced spreading on both surfaces. The mean projected areas of adhered cells on membranes functionalized with poly-HA and oligo-HA calculated from Figure 2A and Figure 2B were comparable, *A*_poly_  =  480 µm^2^ and *A*_oligo_  =  560 µm^2^, respectively. In control experiments, 1AS cells showed almost no sign of adhesion to pure DOPC membranes (Figure 1C), confirming the specificity of adhesion to the HA surfaces.

**Figure 5:**
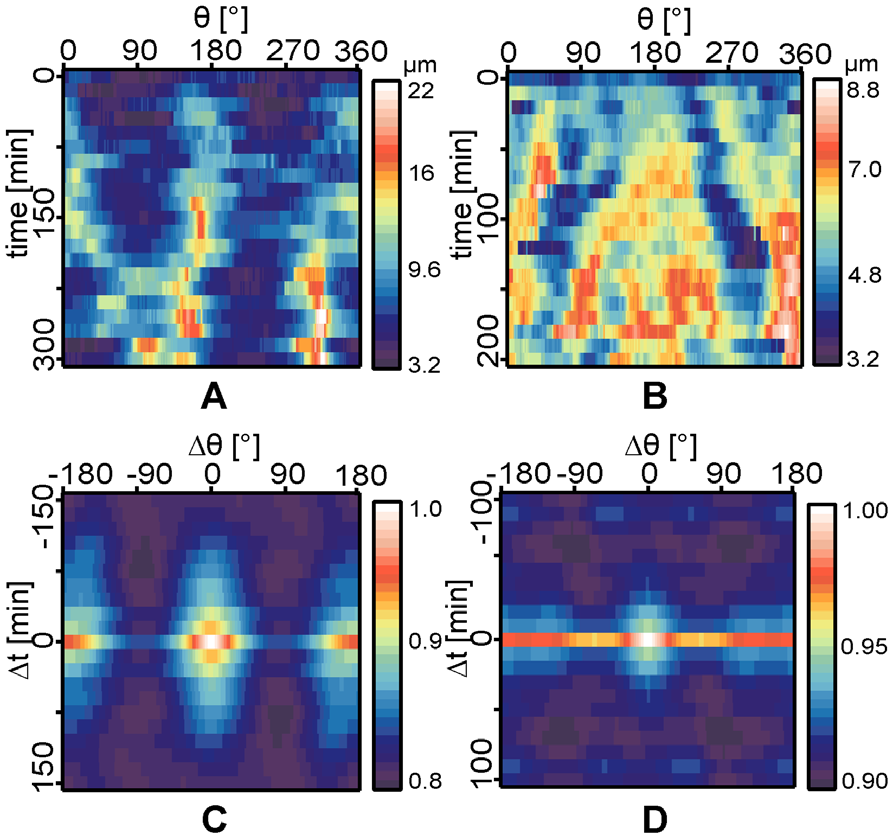
Cell shape fluctuation amplitude and autocorrelation map at early stage. Representative amplitude 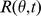 maps of (A) 1AS and (B) ASpSV14 cells plotted as function of *θ* recorded during the early stage of cell adhesion (*t*  =  0–200 min). The corresponding autocorrelation functions for 1AS and ASpSV14 are presented in panel (C) and (D), respectively.

The analysis of the adhesion area, elongation factor and circularity calculated from nine cells (Table 1) suggests that 1AS cells spread isotropic on both poly-HA and oligo-HA surfaces. In fact, regions of tight adhesion identified by low pixel intensities (typically, the separation distance below 10 nm [39]) were found near the cell periphery. We therefore conclude that oligo-HA as well as poly-HA can act as specific ligands for 1AS cells. As we observed no difference in binding of 1AS cells to poly-HA and oligo-HA surfaces, in the subsequent experiments we used exclusively oligo-HA with a narrow size distribution (6–8 disaccharide repeat units).

**Figure 6:**
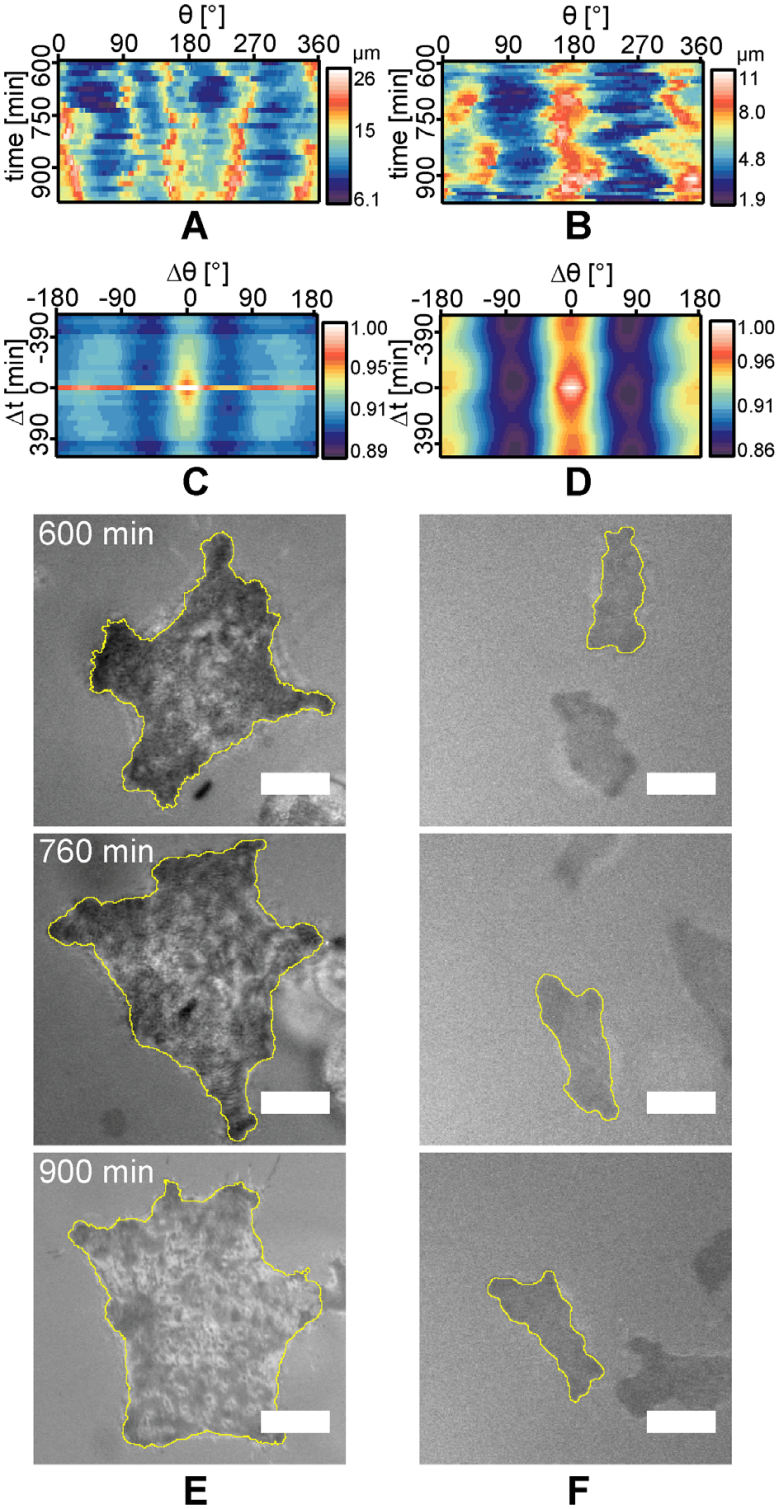
Cell shape fluctuation amplitude and autocorrelation map at late stage. (A) Amplitude 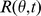 map of representative 1AS and (B) ASpSV14 cells plotted as function of *θ* recorded after the establishment of stable adhesion (*t*  =  600–1000 min). The corresponding autocorrelation of 1AS and ASpSV14 cells are presented in panel (C) and (D), respectively. Three RICM raw images are shown for (E) 1AS and (F) ASpSV14 cells at different time points. Scale bars: 10 µm.

**Figure 7:**
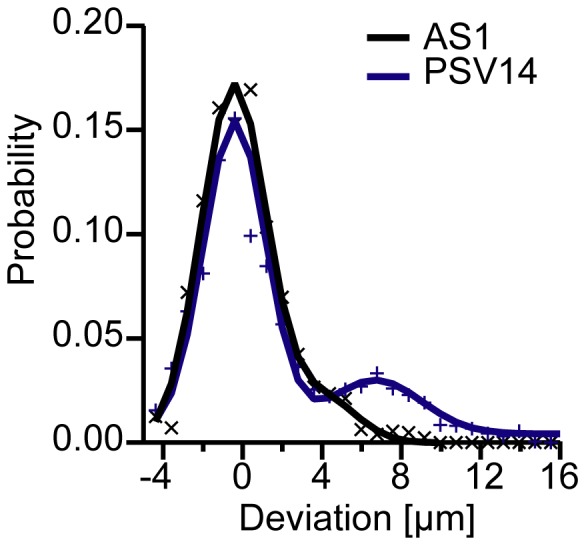
Symmetry break of cells. Degree of cell deformation of a single, representative 1AS cell (black line) and a ASpSV14 cell (blue line) with respect to the direction of cell motility.

Next we examined how the spacing of tethered HA oligosaccharides influences the binding of 1AS cells to these surfaces. The proportion of 1AS cells adhering to the oligo-HA supported membranes for various mean distances between oligo-HA molecules was evaluated as a function of time. Strikingly, only a slight change in <*d*> from 5.5 nm to 11 nm resulted in a significant difference in both the kinetics and efficiency of cell adhesion (Figure 2). At <*d*> ∼ 11 nm, 1AS cells needed 90–120 min to reach saturation levels of adhesion (40–50 %), while at <*d*> ∼ 5.5 nm for the same cells only 30–60 min was required to reach much a higher saturation level of adhesion (> 80 %). These results suggest that specific adhesion of 1AS cells to oligo-HA is highly sensitive to the spatial presentation of the oligo-HA molecules on supported membranes at the level of nm accuracy.

Next we examined the ability of 1AS cells expressing different CD44 isoforms to bind to oligo-HA tethered to supported membranes at <*d*> ∼ 5.5 nm for 4 h. Under these conditions, RICM and confocal fluorescence images were obtained for 1AS cells, AS-K162R166 cells, ASpsV14 cells, and AS-R44 cells (Figure 3). In parallel, we carried out control experiments for all four cell types to examine their adhesion to pure DOPC membranes, and confirmed that non-specific adhesion is negligibly small (<10 %) for all the cell types (data not shown). Prior to the imaging, the cells were fixed, and actin and DNA were stained with Alexa 488 phalloidin and DRAQ5, respectively. For each cell line, between 30 to 90 cells were analyzed, and a minimum of five cells were selected for confocal microscopy. Representative examples are shown in Figure 3. 1AS cells take up a pentagonal or heptagonal shape, and exhibit a more isotropic spreading than the AS-K162R166 and ASpsV14 cells (Figure 3A). In contrast, the AS-R44 cells remained almost spherical (Figure 3D), and the RICM image showed no sign of significant adhesion by these cells to the membrane (Figure 3D, left column).

The presence of a dense meshwork of peripheral actin and the formation of stress fibers as observed for the parental 1AS cells has been associated with cells that are not mobile [40]. On the other hand, AS-K162R166 cells (Figure 3B) and ASpSV14 cells (Figure 3C) cells show characteristic features of highly mobile, migrating cells, such as dense lamellipodial actin at the spreading front (indicated by arrows), and stress fibers along the major axis of the cells. It should be noted that the area of adhesion is comparable between 1AS, AS-K162R166 and ASpSV14 cells, despite the significant differences in cell morphology between 1AS cells and their CD44v4-v7-expressing AS-K162R166 and ASpSV14 derivates (Figure S1).

### Morphological Dynamics of 1AS and ASpSV14 Cells

To highlight the time evolution of cell morphology in response to oligo-HA surfaces, we first extracted the peripheral edge of cells from the time-lapse RICM images. A total of five 1AS and seven ASpSV14 cells were analyzed in this way. Figure 4A shows a representative example of an ASpSV14 cell on a supported membrane functionalized with oligo-HA (<*d*> ∼ 5.5 nm, *t*  =  9 h). After the delineation of the peripheral edge of the cell using the contrast in the pixel values, the radial distance *r* between the edge of the cell (yellow line) and the center of mass was plotted as a polar coordinate (*r*, *θ*), as shown in Figure 4B. Here, *θ*  =  0 is defined as vertical direction in the laboratory system. This enabled us to track the direction of the cell movement and to identify the morphological anisotropy of a cell at time point *t*. The amplitude of the shape fluctuation is defined as 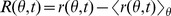, where 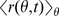 is the mean radial distance over *θ*  =  0–360°. By plotting 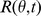 labeled with a color code as a function of angle *θ* over time *t* (Figure 4C), the dynamic morphological changes in the target cell can be depicted. Furthermore, spatio-temporal patterns hidden behind the stochastic noise of morphological dynamics can be extracted by analyzing their correlation functions [32]. The autocorrelation function of the amplitude map can be calculated by:
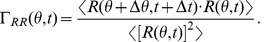
(10)


As shown in Figure 4D, the autocorrelation function calculated from the amplitude map (Figure 4C) suggests that the cell is linearly stretched up to Δ*t* ∼ ± 200 min, contracts for a round contour line at Δ*t* ∼ ± 300 min, and subsequently is linearly stretched in a perpendicular direction after Δ*t* ∼ ± 400 min, which can be identified by a shift of peak positions by ∼ 90°.

Figures 5A and 5B represent the amplitude 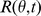 maps of representative 1AS and ASpSV14 cells plotted as a function of *θ* and *t* during the earlier stage of adhesion (*t*  =  0–200 min) to oligo-HA-functionalized membranes at <*d*> ∼ 5.5 nm. As presented in the figure, both cells show very little deviation from the mean radial distance from the center of mass. The corresponding autocorrelations for (C) 1AS and (D) ASpSV14 also show no characteristic feature, suggesting that both metastatic and non-metastatic cells spread isotropically when they are initially in contact with the oligo-HA-functionalized membranes. This experimental finding is consistent with the previous reports on the initial spreading of chondrocytes [41] and fibroblasts [42].

On the other hand, a clear transition in dynamic cell morphology was observed after the cancer cells established stable adhesion. The transition was observed around *t*  =  300 min for both metastatic ASpSV14 and poorly metastatic 1AS cells. After the transition, the cells showed distinct differences in amplitudes and autocorrelations. The 1AS cells exhibited more than 3 distinct maxima with comparable amplitudes of around 20 µm at t  =  750 min (Figure 6A), implying that the adhered cell developed into a hexagonal shape. As the Figure 6A and 6E demonstrate, it is very difficult to distinguish characteristic patterns only from the raw amplitude maps, because the morphological dynamics of biological cells are highly stochastic. Intrinsic stochastic noise from dynamic systems was overcome by calculating the autocorrelation function (Equation 1). As shown in Figure 6C, three stable and pronounced autocorrelation peaks for rotational symmetry at Δ*θ*_max_  =  −120°, 0°, and 120° were identified. The temporal autocorrelation intensity rapidly decayed for Δt ≠ 0 min, suggesting that the 1AS cell rotates without changing its shape.

In contrast, the amplitude map of metastatic ASpSV14 cells showed two pronounced maxima up to *R*_max_ ∼ 11 µm (Figure 6B). The corresponding autocorrelation exhibits two positive correlations separated by ∼ 180° (Δ*θ*_max_  =  0° and ± 180°), which are stable over long Δ*t* (Figure 6D). This indicates that the cell was linearly stretched and moved for more than 2 h in the same direction. RICM images of ASpSV14 cells at fixed image locations (Figure 6F) illustrate the movement and elongation of the cell before changing its orientation. This finding is in contrast to the isotropic spreading observed at earlier time points (Figure 5C and 5D).

### Motility of Cancer Cells Expressing CD44s and CD44v4-v7

In the next step, we studied the motility of ASpSV14 and 1AS cells by tracking the displacement of the center of mass, determined from the projected area of adhesion. Here, the mean velocity of the cell 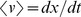 was defined as the displacement of the cell *dx* sampled within a time interval of every *dt*  =  30 min for nine ASpSV14 cells and four 1AS cells. The mean velocity remained almost constant for both cell types over 20 h. The mean velocity of ASpSV14 cells, <*v*_psV14_>  =  5 ± 0.2 µm/h, was only slightly greater than that of 1AS cells, <*v*_1AS_>  =  4 ± 0.2 µm/h, where errors represent the standard deviation of mean values.

To deduce the relationship between the direction of motility and the morphological change, we also calculated the probability distribution function of the degree of cell deformation and the direction of cell motility 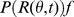:
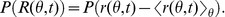
(14)


Note that *θ*  =  0 is defined as the direction of the cell motility. The velocity and deformational change of cancer cells was evaluated for six 1AS and four ASpSV14 cells. As presented in Figure 7, a clear sign of the break of symmetry for a single, representative ASpSV14 cell can be identified as a distinct sub-peak that deviates from the center by 8 µm in the direction of cell motility. This is in contrast to that of 1AS cells possessing the peak near the center, which corresponds to random, isotropic movement. For both cell lines, the histogram was calculated for more than 55 cell contour lines.

## Discussion

Although the interaction of CD44 with HA is clearly important in the context of cancer [4], the role of these molecules in tumor growth and metastasis needs further investigation to understand the sometimes contradictory results regarding the nature of this interaction. In particular, analysis using defined spacing of HA of a homogeneous size has been lacking to date. Here we have used HA tethered to supported membranes to investigate biophysical parameters associated with cell binding to HA. We found that while CD44s-expressing 1AS cells bound equally well to poly-HA and oligo-HA surfaces, binding was exquisitely sensitive to the spatial distribution of HA. At the single cell level, ectopic expression of CD44v4-v7 in these cells did not alter initial binding to the oligo-HA surfaces, but later induced altered cell morphology and enhanced directional migration.

Previously we have shown that in contrast to ASpSV14 cells, 1AS cells bind very poorly to immobilized HA surfaces [26]. In these cell binding assays, absorption of HA to plastic surfaces was employed, where no control over the density or spatial distribution of the HA molecules was possible [26]. However, in this present study, both cell types bound to oligo-HA tethered membranes, although ASpSV14 cells responded differently in terms of morphology and motility after initial binding to the surfaces. We also found that the binding of 1AS cells to oligo-HA surfaces was strongly influenced by the spacing of the HA molecules. It is therefore possible that the spatial distribution of HA in previous assays was not optimal for binding of 1AS cells, but sufficed for binding of ASpSV14 cells that express in addition the CD44v4-v7 protein. Further work will be required to determine whether ASpSV14 cells are less sensitive to the spatial distribution of HA than 1AS cells. This would seem likely, due to the clustering of CD44 on the surface of the cells that leads to enhanced HA binding properties [9].

The 1AS derivative AS-R44 ectopically expresses a mutant form of CD44v4-v7 that cannot bind to HA [26]. Surprisingly, although AS-R44 cells express CD44s similar to the parental 1AS cells that can bind to oligo-HA surfaces, the AS-R44 cells were unable to interact with these surfaces (Figure 3). These data therefore suggest that the non-HA-binding mutated form of CD44v4-v7 may affect the HA binding activity of the endogenous CD44s protein. Consistent with this notion, the AS-K162R166 cells that express a mutated form of CD44v4-v7 that shows only a partially reduced binding capacity to HA [26] behaved similarly in the binding assays to the ASpSV14 cells that express the wild-type CD44v4-v7 protein.

Under the spatial distribution of oligo-HA employed in this study, the mean translocation velocity of ASpSV14 cells (<*v*_psV14_>  =  5.0 ± 0.2 µm/h) was comparable to that of 1AS cells (<*v*_1AS_>  =  4.4 ± 0.2 µm/h) over 20 h, despite a significant difference in their morphological dynamics (Figure 6). However, analysis of directed cell deformation (Figure 7) suggests that while 1AS cells only randomly migrate on the oligo-HA surface, ASpSV14 cells exhibit enhanced directional motility. These differences may reflect the highly metastatic behaviour of ASpSV14 cells *in vivo* compared to the weak metastatic behaviour of 1AS cells [7], [16]. Furthermore, although 1AS cells exhibit cytoskeletal features previously associated with immobile cells [40], the fact that their velocity was observed to be similar to ASpSV14 cells suggests that these features rather reflect the dynamic morphological characteristics of the cells.

In this study, we utilized quantitatively functionalized supported membranes and studied the adhesion, morphological patterns, and motility of pancreatic adenocarcinoma cells expressing different CD44 isoforms. To discriminate the characteristic spatio-temporal patterns from the stochastic noise from dynamic cell systems, we introduced a relatively simple but straightforward statistical image analysis such as autocorrelation functions. Previously, Maeda et al. investigated the morphology and orientational correlation of slime mold *Dictyostelium discoideum* on glass substrates [32], reporting that *D. discoideum* undergoes stochastic transitions within a time window of 10–20 min. Our results suggest that the morphological dynamics of pancreatic cancer cells is much slower, with characteristic time windows of several hours. This can be attributed in part to the prominent spreading and adhesion of cancer cells mediated via specific HA-CD44 interactions, and may require both adhesion to the surface (grip) and successive cleavage of CD44 (release) during cell migration as suggested by a previous study [43].

The dynamics of “self-propelled” particles is attracting increasing attention in the field of statistical physics far from equilibrium. If one considers the motility of biological cells, the motion of the center of mass is caused by shape deformation. Recently, Ohta and Sano proposed two approaches to describe the dynamic coupling between motion and deformation in two-dimensional space [44]. One is called as a “tensor model” which describes the velocity of center of mass by two tensor variables for deformation based on symmetry consideration, while the other is represented in the form of a partial differential equation for a Euclidean invariant variable of a closed loop. The statistical analysis we introduced here would provide a powerful tool to obtain the spatio-temporal patterns of cancer cells on the functionalized surface, which would further enable us to correlate the mode of motion (e.g. translation and rotation) and the mode of shape deformation (e.g. hexagonal, linear, etc.).

In summary, the combination of quantitatively functionalized surfaces and statistical image analysis provides a basis for gaining spatio-temporal pattern formation in pancreatic cancer cells adhered via CD44-HA interactions. Our initial findings provide the basis for further studies to examine how the dynamic morphology and the motion of cancer cells are modulated in a CD44 isoform-dependent manner.

## Supporting Information

Figure S1 **Average area of adhesion.** Average area of adhesion after 3 h incubation of 1AS cells, AS-K162R166 cells, ASpSV14 cells, and AS-R44 cells on oligo-HA substrates at <*d*> ∼ 5.5 nm. More than 50 cells were measured for each cell type and standard deviation is given as error bar.
(TIF)
