# Supplementary figures and images for: Spatio-Temporal Patterns of Pancreatic Cancer Cells Expressing CD44 Isoforms on Supported Membranes Displaying Hyaluronic Acid Oligomers Arrays
Source: PLoS One. 2012 Aug 14;7(8):e42991. doi: 10.1371/journal.pone.0042991 (PMC3419250; doi:10.1371/journal.pone.0042991)

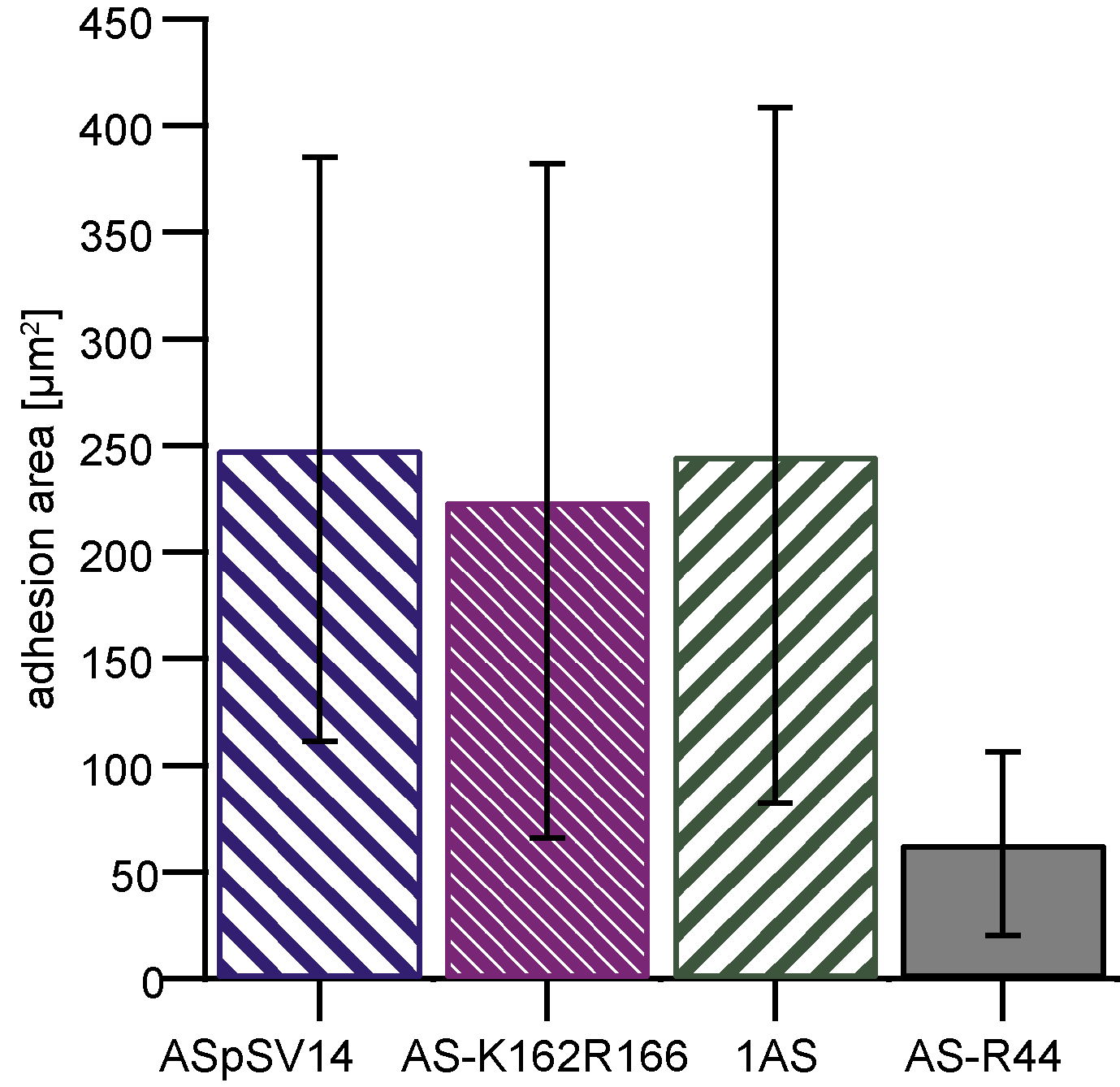

Supplement: Figure S1 — Average area of adhesion. Average area of adhesion after 3 h incubation of 1AS cells, AS-K162R166 cells, ASpSV14 cells, and AS-R44 cells on oligo-HA substrates at ∼ 5.5 nm. More than 50 cells were measured for each cell type and standard deviation is given as error bar. (TIF) [file pone.0042991.s001.tif]
